# Inter-Visit Reliability of Smooth Pursuit Neck Torsion Test in Patients with Chronic Neck Pain and Healthy Individuals

**DOI:** 10.3390/diagnostics11050752

**Published:** 2021-04-22

**Authors:** Ziva Majcen Rosker, Miha Vodicar, Eythor Kristjansson

**Affiliations:** 1 Faculty of Sport, University of Ljubljana, 1000 Ljubljana, Slovenia; 2 Department of Orthopaedic Surgery, University Medical Centre, 1000 Ljubljana, Slovenia; miha.vodicar@kclj.si; 3 Landspitali University Hospital, 101 Reykjavik, Iceland; eythork@simnet.is

**Keywords:** smooth pursuit neck torsion test, neck pain, reliability, oculomotor functions

## Abstract

Visual disturbances are commonly reported in patients with neck pain. Smooth pursuit neck torsion (SPNT) test performed in neutral position and with trunk rotated under the stationary head has been used to discriminate between those with cervical component and those without. However, no studies investigated the reliability of the SPNT-test in patients with chronic neck pain and healthy controls. The aim of this study was to assess inter-visit reliability of the SPNT-test while applying different amplitudes and velocities of target movement. Thirty-two controls and thirty-one patients were enrolled in the study. The SPNT-test was performed in neutral position and through 45° torsion positions. The test was performed at 20°/s, 30°/s and 40°/s velocities and at 30°, 40° and 50° amplitudes of cyclic sinusoidal target movements. Interclass correlation coefficient and smallest detectable change were calculated for parameters of gain and SPNT-differences. In patients, moderate to good reliability was observed for gain at 40° and 50° amplitudes and for 20°/s and 30°/s velocities, while moderate to excellent reliability for gain was observed in controls. Both groups presented with moderate to good reliability for SPNT-difference. Our findings imply that amplitudes of 40° and 50° and velocities of 20°/s and 30°/s are the most reliable and should be applied in future studies assessing oculomotor functions during the SPNT test.

## 1. Introduction

According to research and anecdotal evidence, patients with chronic neck pain frequently complain about different characteristics of visual disturbances such as blurred vision, words jumping on the page and difficulty focusing and concentrating on reading [1]. These could be due to malfunctions of the oculomotor system that enable them to efficiently direct and keep their gaze on a slowly moving target [2].

An important component of the oculomotor system is smooth pursuit eye movements [3]. The task is often used to assess eye movement control with participants instructed to follow a horizontally moving target. In patients with neck pain, a proposed mechanism for disturbances in smoothly following the target is likely to be a mismatch between cervical proprioceptive input and its interconnection to the vestibular and the visual systems [4]. Disturbed afferent input from the cervical spine can lead to less accurate image stabilization of the moving target on or near the fovea, especially when the neck is in the torsioned position [5].

The SPNT test was first introduced in 1998 by Tjell and Rosenhall [6]. The test is performed in neutral and rotated positions (i.e., to the left and right sides, respectively) by rotating the trunk underneath the stationary head in order to recruit neck proprioception but not the vestibular mechanoreceptors. Consequently, the SPNT test is positive when the ability to track a horizontally moving target is decreased in rotated positions as compared to neutral position.

Since it was introduced the SPNT test has been frequently used to screen for deficiencies in eye movement control as a consequence of altered afferent input derived from cervical spine resulting in altered proprioceptive reflexes of the neck, the cervico-collic and cervico-ocular reflexes. Abnormal values of the test typically indicate error in proprioceptive information derived from the neck, transmitted by these reflexes. Over the years, many attempts have been made to elucidate deficits in oculomotor performance in patients with neck pain, but results remain inconclusive. Some researchers managed to find differences between patients and healthy individuals [7], while others were unable to make the same conclusion [8]. Possible reasons as to why inconsistencies exist could be the lack of methodological consensus in equipment used (i.e., electro-oculography vs. video-oculography), application of chin rest in some studies, different neck torsion positions used and automated or semi-automated analysis of eye movement data, all of which could possibly influence the results [7,8,9,10]. In addition, different target velocities and amplitudes were applied across studies, with velocities ranging from 20 [10] to 37°/s [9] and higher velocities used in healthy subjects [11]. Similarly, different amplitudes were applied while tracking a horizontally moving target [7,10], suggesting non-homogenous approaches while assessing oculomotor functions. Discrepancy in the literature due to methodological inconsistencies could possibly result in differences in sensitivity.

Surprisingly, to our knowledge there is no research assessing test-retest reliability of the SPNT test which is a crucial component when reporting diagnostic validity. Smooth pursuit eye movement test has been shown to range from moderate [11] to good reliability [12] in healthy individuals, but the test was only conducted in a neutral position. As the SPNT test is commonly applied in healthy subjects [13] and in patients with chronic neck pain [10], it would be of importance to assess inter-visit reliability while applying different target velocities and amplitudes in neutral and neck torsioned positions. Therefore, the aim of this research was to study inter-visit reliability of the SPTN test at different amplitudes, velocities and in neutral as well in torsion positions for healthy individuals and patients with chronic neck pain.

## 2. Materials and Methods

### 2.1. Participants

Sixty-four participants, of which thirty-two were patients with chronic neck pain (23 women and 9 men; average age 46.2 ± 4.8 years, range 27–53 years, average pain duration 13.6 ± 8.3 months) and thirty-two healthy individuals (19 women and 13 men; average age 37.8 ± 6.1 years, age range 23–49 years) participated in the study. Healthy participants were recruited among university staff, doctoral students and their friends. Patients with neck pain were referred by an orthopedic surgeon and were previously assessed for suitability via a telephonic interview. Each patient underwent an MRI, indicating some sort of lower cervical spine degenerative structural abnormality (disc protrusions or herniations at the levels from C5 to Th1, facet joints edema at the levels from C5 to Th1, low grade spondylolisthesis and cervical spinal stenosis). Inclusion criteria for both groups were age range between 18 and 55 years. Subjects with chronic idiopathic neck pain having had to experience pain in the neck from 6 months to 5 years, were required to have at least 50 degrees of cervical rotation to both sides, a minimum score of 36 on the Dizziness Handicap Inventory out of 100 and a minimum score of 5 out of 10 on pain visual analogue scale (VAS). The VAS scale was a 10 cm horizontal line with ends marked “no pain” (left) and the “worst pain imaginable” (right) [14,15]. All subjects had to be free from previous injury to the neck or head, shoulder or upper extremities pain, any neurological or vestibular disorders, present with no myelopathy and were required to take no medication or alcohol for the last 30 h prior to participating in the study. Participants were not included in the study in a case of corrected vision (e.g., use of glasses or contact lenses). All participants were required to read and sign a consent form. The study was approved by the national medical ethics committee (number: 0120–47/2020/6) and was performed in accordance to declaration of Helsinki.

### 2.2. Apparatus

A 100 Hz eye tracking device (Pro Glassems 2, Tobii, Danderyd, Sweden) was used to measure and record eye movements during smooth pursuit tasks. Prior to the experiment, a single target calibration routine was performed in the Tobii Pro Glasses Controller (Tobii Pro Glasses Controller, Tobii, Danderyd, Sweden). Participants were required to track a red dot target (size 0.5° of visual angle) projected on a white screen using a projector (Optoma ML1050ST LED Projector, Fremont, CA, USA) 150 cm away at eye level. Subjects were sitting on a custom-made rotatable chair with upper body fixed to the back support. Hip angle was 80° of flexion, while their feet were placed flat on the floor. Measurements were conducted by the same examiner.

### 2.3. Experiment

Patients and healthy subjects answered Dizziness Handicap Inventory and were required to fill out a VAS of pain intensity. The testing protocol consisted of 3 different chair positions: (1) facing forward position (their trunk and head were in a neutral position of 0° in the transverse plane), (2) eccentric rotation of the neck of 45° to the left and (3) to the right, respectively. The order of chair rotations was pseudo-randomized across subjects. In the neutral position the chair was aligned so that the participant was facing forward to the middle of the screen. During trunk rotation their head was in a neutral position while their trunk and lower body were rotated. All tests were performed in an isolated room with dim light.

Before the test, all subjects performed 5 familiarization warm up cycles. For each condition subjects were required to track 10 cycles of cyclic sinusoidal target movements with 60 s rest intervals. Subjects were tested at 3 different maximal target speeds (20°/s, 30°/s and 40°/s) and 3 different amplitudes of 30°, 40° and 50° in all 3 different chair positions, namely, neutral and rotation left and right, respectively. All tasks were performed in a random order. Each chair rotation was followed by a 5 min rest and a recalibration of the eye-tracking device. Participants completed the second oculomotor assessments with an average test-retest interval of 4 d (range 2–13 d).

### 2.4. Data Analysis

The eye movement data were filtered for blinks, saccades and fixations using Tobii Pro Lab software (Tobii Pro lab 1.145, Tobii, Danderyd, Sweden). The square waves (saccades directed counter to each other and having an interval of relative standstill) were removed from the eye movement data using custom-written software in Matlab (R2017b, MathWorks, Natick, MA, USA). The eye movement data were fitted with a corresponding reference sinusoid with synchronized signal acquisition starting points. Each fitted sinusoid consisted of 10 cycles with correspondingly fixed amplitude (converted from angular degrees to pixels) and frequency that matched the profile for each individual condition. The horizontal eye movements were analyzed using gain, calculated as the ratio between fitted eye velocity amplitude and visual target velocity amplitude as described by Tjell et al. [5]. Average gain from the 6th to 9th cycle from each task was used for reliability calculations. In addition, smooth pursuit neck torsion difference (SPNTdiff) was calculated as presented in Equation (1). The calculation was adapted and is similar to that described by Tjell et al. [5]

SPNTdiff = Gain neutral − (Gain torsion L + Gain torsion R)/2(1)

Equation (1) gain neutral represents the average gain in the neutral position from the 6th to 9th cycle, Gain torsion L represents the average gain during the left neck torsion position from the 6th to 9th cycle and Gain torsion R represents the average gain during the right neck torsion position from the 6th to 9th cycle.

### 2.5. Statistical Analysis

Statistical analysis was performed in SPSS (SPSS 23.0 software, SPSS Inc., Chicago, IL, USA). Descriptive statistics (average and standard deviation) were calculated. Normality of distribution was analyzed separately using the Shapiro–Wilk test for each condition at each visit. A two-way mixed effect intraclass correlation coefficient for absolute agreement (ICC(3.1)) and a 95% confidence interval were used to ascertain reliability [16]. An ICC < 0.5 was treated as poor reliability, ICC 0.5–0.75 as moderate reliability, ICC 0.75–0.9 as good reliability and ICC > 0.9 as excellent reliability [17]. In addition, coefficient of variation (CV), standard error of measurement and smallest detectable change group (SDCg) were calculated.

## 3. Results

In total, 32 healthy subjects and 32 patients with chronic neck pain were included for the inter-visit reliability study. However, one of the patients with chronic neck pain decided not to continue with further assessment after the first visit. Gain and SPNTdiff parameters were normally distributed in all conditions observed.

### 3.1. Gain in Healthy Controls

The results of the reliability analysis for the healthy subjects are presented in Table 1. The ICC proved to vary from moderate to excellent. In general, the ICC was higher for the neutral position as compared to trunk rotated positions. The highest ICC (moderate to excellent) was observed for the 40° and 50° amplitude of target movement and at velocities of 20°/s and 30°/s. The lowest (moderate) reliability was observed for the 30° amplitude of target movement regardless of the target movement velocity. The SDCg ranged from 0.019 to 0.043 and did not show any changes with regard to the amplitude or velocity of the target movement.C^1^

### 3.2. Gain in Patients with Neck Pain

Table 2 presents the results of the reliability analysis for the group of neck pain patients. In this group the ICC varied from moderate to good and showed similar trends as in the healthy group. The most reliable conditions were the eye movement amplitudes of 40° and 50°, especially the 50° amplitude. The neutral position showed somewhat higher ICC compared to the trunk rotated positions, except for the condition of 50° amplitude and velocity of 20°/s. The SDCg was higher as in control group ranging from 0.028 to 0.083 and with no specific trend of increase or decrease regarding the amplitude and velocity of eye movement.

### 3.3. SPNT Difference

Results of the reliability study for the SPNTdiff in both groups are presented in Table 3. In general, the ICC of SPNTdiff for both groups was lower (moderate to good) as compared to ICC for gain. In general, the ICC was higher for the control group as compared to the neck pain patient group. Similarly, ICC was higher at 40° and 50° amplitudes.

## 4. Discussion

Based on the results, it can be concluded that overall there is moderate to good inter-visit reliability for average gain in most conditions for both groups as presented in Table 1 and Table 2. SPNTdiff showed similar results with moderate and good reliability achieved for majority of conditions where patients with neck pain presented slightly lower ICC (Table 3). Results of our study show specific trends that could indicate amplitude rather than velocity as being more differentiating when reporting on the level of reliability. In addition, SDCg was higher in patients than in healthy controls, however, this was not related to amplitude or velocity of the target movement.

Most conditions with 50° amplitude of target movement showed superior reliability in both groups. This could be partially explained by the functional connections between extraocular and cervical muscle activity. In the study by Bexander and Hodges [18], neck muscle activity was investigated during eye movements in healthy subjects and in patients with whiplash associated disorders (WAD). Their findings imply that healthy subjects present with bilateral activation of obliquus capitis inferior (OI) when their eyes are moving in each direction while keeping their head in a neutral position. The OI is an important stabilizer of the atlanto-axial joint that contributes towards the first 45° of head rotation [19]. Moreover, Bexander and Hodges [18] report greater OI activity with increase in eye movement amplitude, suggesting higher co-contraction, possibly increasing stiffness and sensory feedback. Increased stiffness would generally lead to a more stable system and less spontaneous oscillations [20,21]. The latter could indicate more accurate control over head and neck posture providing less mechanical and sensory noise to the eye movements. This could possibly lead to higher reliability in the SPNT test which was in accordance with the results from our study where reliability of the SPNT test was superior at 50° amplitude and the lowest at 30° amplitude.

Results from our study show that neck pain patients had slightly poorer reliability than healthy individuals, but showed a similar trend where 50° amplitude of the SPNT test was most reliable. In addition to amplitude, eye movement velocity gives some further insights into reliability characteristics. Interestingly, both groups from our study presented with highest reliability in a neutral position when the target was moving at 30°/s that slightly increased when the trunk was rotated, which is in line with the above hypothesis. Similar findings about the reliability of the target movement velocity were found in the study by Ettinger et al. [11]. Both groups in our study showed highest gain at 20°/s, which is in line with the results from the study by Schalen et al. [22], however, this was not an indicator for superior reliability in our study.

In the study by Bargary et al. [12] reliability throughout three different speeds was assessed in healthy individuals, however, only overall reliability was reported. Their results presented slightly higher reliability than the results from our study. One of the reasons could be the use of a chin rest in their study. The latter is desirable and frequently used when assessing patients with neurological and psychological deficits [23,24] with the focus on excluding all external influences other than the observed pathology. However, these laboratory settings exclude possible head oscillations that are present in everyday tasks and could influence eye movement control [25]. An important contributor to accurate eye movement tracking and visual image stabilization is sufficient stability of the head [26]. It is well researched that patients with neck pain present with poor stability of the head and neck. In cases where a chin rest is used, impairments of the cervical spine afferent input and its effect on oculomotor control are less visible [27].

According to the study by Treleaven et al. [10], pain in the neck was not the main contributor to altered oculomotor functions. In their study, subjective symptoms of dizziness and unsteadiness related more to the deficits in eye movement control. These symptoms are suggested to be a result of sensory mismatch, commonly seen in those with cervical spine involvement. The limitation of our study was that the level of dizziness was not considered in the analysis, however, a cut-off score of at least 36 on the Dizziness Handicap Inventory out of 100 was required for enrolment of patients with neck pain in this study. Dizziness is suggested to be related to sensory impairments, therefore other neck pain disorder pathologies were not considered in our study, such as myelopathy [28,29,30], should be investigated in future studies in relation to eye movement control.

An additional parameter investigated in this study was SPNTdiff. Based on the results, inter-visit reliability for SPNTdiff was lower than for gain in different conditions. In the patient group, reliability of SPNTdiff was moderate, while healthy controls presented with moderate to good ICC. These results were expected as SPNTdiff is calculated from average gain in neutral and in trunk rotated positions. By including three different parameters with their own errors of measurement, the cumulative error could increase and consequently weaken reliability. The most reliable conditions in both groups were at velocities of 20°/s and 30°/s and at amplitudes of 40° and 50°. This is in line with the above observations describing possible factors contributing to reliability of gain. As SPNTdiff is calculated for both sides and described as percentage of difference, possible unilateral structural abnormalities could influence results not considered in our study.

Additionally, SDCg for gain was lower in healthy controls and higher in group of patients with chronic neck pain with no differences between conditions. Future studies should implement SDCg in smooth pursuit sensitivity studies comparing different groups of patients and healthy controls or effects of rehabilitation interventions. A similar trend was observed for SPNTdiff in patients with neck pain presenting with higher SDCg.

## 5. Conclusions

Although an attempt was made to recruit a homogenous group of patients, neck pain disorders have heterogenous pathologies with interconnected signs and symptoms. Therefore, reliability of the SPNT test might vary across different neck pain pathologies. Future studies should include the most reliable parameters and use them for further assessments of the most sensitive parameters across different traumatic and non-traumatic neck pain incidences.

## Figures and Tables

**Table 1:** Average gain reliability for each task in healthy subjects.

Amplitude (°)	Velocity (°/s)	Position	ICC ^1^	CI ^2^	CV ^3^	SEM ^4^	SDCg ^5^	Mean 1 ^6^	Mean 2 ^7^	SD 1 ^8^	SD 2 ^9^
30	20	N	0.759	0.541–0.925	0.018	0.009	0.022	0.952	0.933	0.013	0.015
30	20	L45	0.618	0.562–0.858	0.019	0.015	0.031	0.945	0.951	0.014	0.016
30	20	R45	0.723	0.607–0.834	0.018	0.021	0.038	0.948	0.966	0.013	0.016
30	30	N	0.776	0.621–0.847	0.013	0.012	0.034	0.922	0.937	0.010	0.010
30	30	L45	0.778	0.642–0.944	0.016	0.017	0.042	0.938	0.911	0.014	0.013
30	30	R45	0.761	0.508–0.884	0.014	0.016	0.037	0.906	0.919	0.012	0.012
30	40	N	0.681	0.557–0.822	0.019	0.011	0.031	0.889	0.902	0.012	0.015
30	40	L45	0.624	0.344–0.701	0.016	0.016	0.029	0.864	0.893	0.015	0.013
30	40	R45	0.596	0.403–0.694	0.017	0.022	0.041	0.875	0.841	0.016	0.012
40	20	N	0.764	0.654–0.853	0.015	0.008	0.019	0.946	0.977	0.013	0.012
40	20	L45	0.752	0.477–0.875	0.014	0.015	0.027	0.968	0.971	0.011	0.012
40	20	R45	0.757	0.632–0.826	0.015	0.023	0.034	0.940	0.962	0.012	0.013
40	30	N	0.813	0.617–0.924	0.013	0.013	0.029	0.939	0.968	0.011	0.010
40	30	L45	0.783	0.667–0.907	0.014	0.017	0.024	0.919	0.954	0.013	0.012
40	30	R45	0.802	0.684–0.955	0.018	0.016	0.041	0.922	0.960	0.012	0.013
40	40	N	0.753	0.606–0.891	0.018	0.010	0.031	0.906	0.922	0.016	0.014
40	40	L45	0.697	0.534–0.784	0.017	0.024	0.043	0.887	0.903	0.014	0.015
40	40	R45	0.737	0.502–0.901	0.020	0.017	0.026	0.895	0.916	0.015	0.015
50	20	N	0.863	0.554–0.787	0.012	0.007	0.024	0.971	0.965	0.010	0.011
50	20	L45	0.801	0.641–0.911	0.013	0.022	0.036	0.944	0.959	0.010	0.012
50	20	R45	0.761	0.531–0.839	0.011	0.014	0.040	0.936	0.924	0.009	0.010
50	30	N	0.9	0.832–0.980	0.013	0.010	0.033	0.968	0.951	0.010	0.011
50	30	L45	0.908	0.815–0.979	0.014	0.028	0.027	0.945	0.962	0.013	0.012
50	30	R45	0.871	0.811–0.937	0.017	0.021	0.031	0.960	0.943	0.012	0.014
50	40	N	0.773	0.586–0.892	0.019	0.013	0.025	0.897	0.914	0.015	0.016
50	40	L45	0.83	0.647–0.962	0.020	0.017	0.031	0.883	0.905	0.014	0.016
50	40	R45	0.693	0.337–0.924	0.016	0.020	0.028	0.903	0.921	0.016	0.014

^1^ ICC—intraclass correlation coefficient; ^2^ CI—95% confidence interval; ^3^ CV—coefficient of variation; ^4^ SEM—standard error of measurement; ^5^ SDCg—smallest detectable change of a group; ^6^ Mean 1—group average at the first visit; ^7^ Mean 2—group mean at the second visit; ^8^ SD 1—group standard deviation at the first visit; ^9^ SD 2—standard deviation at the second visit.

**Table 2:** Average gain reliability for each task in chronic neck pain patients.

Amplitude (°)	Velocity (°/s)	Position	ICC ^1^	CI ^2^	CV ^3^	SEM ^4^	SDCg ^5^	Mean 1 ^6^	Mean 2 ^7^	SD 1 ^8^	SD 2 ^9^
30	20	N	0.691	0.421–0.751	0.025	0.011	0.032	0.830	0.817	0.018	0.019
30	20	L45	0.543	0.324–0.708	0.026	0.035	0.067	0.784	0.821	0.017	0.018
30	20	R45	0.594	0.440–0.752	0.032	0.028	0.064	0.761	0.745	0.016	0.023
30	30	N	0.739	0.653–0.888	0.024	0.015	0.039	0.696	0.724	0.021	0.016
30	30	L45	0.694	0.433–0.788	0.019	0.024	0.079	0.658	0.666	0.019	0.011
30	30	R45	0.701	0.612–0.775	0.022	0.037	0.083	0.673	0.727	0.024	0.012
30	40	N	0.758	0.622–0.874	0.032	0.010	0.028	0.703	0.686	0.020	0.021
30	40	L45	0.647	0.482–0.810	0.041	0.027	0.065	0.713	0.623	0.019	0.025
30	40	R45	0.606	0.457–0.723	0.045	0.042	0.058	0.736	0.595	0.014	0.022
40	20	N	0.762	0.640–0.851	0.014	0.014	0.040	0.798	0.805	0.014	0.011
40	20	L45	0.753	0.527–0.884	0.025	0.024	0.071	0.766	0.793	0.014	0.017
40	20	R45	0.751	0.600–0.841	0.023	0.019	0.069	0.774	0.791	0.019	0.018
40	30	N	0.788	0.594–0.902	0.226	0.012	0.033	0.720	0.735	0.014	0.016
40	30	L45	0.765	0.630–0.842	0.046	0.034	0.082	0.705	0.677	0.017	0.029
40	30	R45	0.757	0.651–0.924	0.031	0.031	0.067	0.691	0.700	0.018	0.021
40	40	N	0.733	0.522–0.834	0.045	0.007	0.036	0.639	0.629	0.017	0.028
40	40	L45	0.662	0.472–0.767	0.042	0.026	0.072	0.637	0.617	0.019	0.025
40	40	R45	0.581	0.389–0.656	0.033	0.029	0.068	0.631	0.621	0.013	0.019
50	20	N	0.744	0.609–0.833	0.021	0.012	0.029	0.837	0.820	0.013	0.015
50	20	L45	0.88	0.768–0.917	0.018	0.024	0.073	0.829	0.828	0.012	0.013
50	20	R45	0.864	0.783–0.950	0.015	0.022	0.064	0.809	0.818	0.013	0.009
50	30	N	0.879	0.831–0.940	0.042	0.090	0.036	0.737	0.716	0.025	0.030
50	30	L45	0.801	0.715–0.879	0.018	0.019	0.055	0.753	0.748	0.017	0.012
50	30	R45	0.774	0.622–0.861	0.030	0.025	0.069	0.613	0.658	0.024	0.017
50	40	N	0.759	0.652–0.876	0.023	0.013	0.030	0.669	0.670	0.017	0.016
50	40	L45	0.684	0.548–0.792	0.032	0.034	0.066	0.684	0.693	0.021	0.022
50	40	R45	0.735	0.576–0.874	0.031	0.032	0.075	0.614	0.593	0.012	0.018

^1^ ICC—intraclass correlation coefficient; ^2^ CI—95% confidence interval; ^3^ CV—coefficient of variation; ^4^ SEM—standard error of measurement; ^5^ SDCg—smallest detectable change of a group; ^6^ Mean 1—group average at the first visit; ^7^ Mean 2—group mean at the second visit; ^8^ SD 1—group standard deviation at the first visit; ^9^ SD 2—standard deviation at the second visit.

**Table 3:** Reliability of smooth pursuit neck torsion difference for both groups.

	Amplitude	Velocity (°/s)	ICC ^1^	CI ^2^	CV ^3^	SEM ^4^	SDCg ^5^	Mean 1 ^6^	Mean 2 ^7^	SD 1 ^8^	SD 2 ^9^
Patients	30	20	0.642	0.417–0.804	1.467	0.018	0.051	0.056	0.011	0.082	0.069
	30	30	0.638	0.503–0.744	2.078	0.033	0.091	0.073	–0.014	0.080	0.074
	30	40	0.589	0.343–0.733	1.836	0.043	0.118	0.052	0.013	0.103	0.091
	40	20	0.746	0.437–0.811	1.283	0.038	0.107	–0.067	0.045	0.068	0.054
	40	30	0.751	0.494–0.819	2.856	0.018	0.049	0.001	0.006	0.046	0.057
	40	40	0.497	0.181–0.682	2.524	0.021	0.059	–0.021	0.092	0.100	0.067
	50	20	0.762	0.597–0.901	2.281	0.039	0.075	–0.031	0.046	0.107	0.122
	50	30	0.759	0.532–0.906	−5.233	0.048	0.133	–0.026	–0.037	0.119	0.126
	50	40	0.673	0.510–0.821	1.460	0.030	0.083	0.065	0.086	0.099	0.139
Healthy	30	20	0.689	0.504–0.916	1.322	0.014	0.015	0.004	0.009	0.077	0.062
	30	30	0.721	0.578–0.879	1.507	0.025	0.020	0.010	0.001	0.064	0.058
	30	40	0.701	0.527–0.821	2.013	0.037	0.051	0.040	–0.009	0.086	0.062
	40	20	0.812	0.699–0.972	1.750	0.022	0.017	0.002	0.005	0.042	0.065
	40	30	0.781	0.517–0.905	1.406	0.034	0.022	0.005	–0.004	0.059	0.080
	40	40	0.683	0.521–0.783	1.943	0.017	0.009	0.010	0.004	0.072	0.094
	50	20	0.78	0.685–0.921	–1.447	0.019	0.010	–0.001	–0.010	0.096	0.075
	50	30	0.773	0.573–0.961	1.612	0.041	0.025	0.008	–0.001	0.092	0.101
	50	40	0.751	0.494–0.887	1.818	0.023	0.019	0.002	0.001	0.073	0.097

^1^ ICC—intraclass correlation coefficient; ^2^ CI—95% confidence interval; ^3^ CV—coefficient of variation; ^4^ SEM—standard error of measurement; ^5^ SDCg—smallest detectable change of a group; ^6^ Mean 1—group average at the first visit; ^7^ Mean 2—group mean at the second visit; ^8^ SD 1—group standard deviation at the first visit; ^9^ SD 2—standard deviation at the second visit.

## Data Availability

The data presented in this study are available on request from the correspondence author.
